# Cytomegalovirus and Epstein-Barr Virus Co-infection in a Patient With Chronic Granulomatous Disease Co-existing With Familial Mediterranean Fever and Early-Onset Inflammatory Bowel Disease: A Case Report

**DOI:** 10.7759/cureus.44360

**Published:** 2023-08-30

**Authors:** Yazan O Al Zu’bi, Ahmed H Al Sharie, Suleimman Al-Sweedan, Sohaib Al-Khatib, Saied A Jaradat, Eyad Al Tamimi

**Affiliations:** 1 Department of Pediatrics, Faculty of Medicine, Jordan University of Science and Technology, Irbid, JOR; 2 Department of Pathology and Microbiology, Faculty of Medicine, Jordan University of Science and Technology, Irbid, JOR; 3 Department of Biotechnology and Genetic Engineering, Faculty of Science and Arts, Jordan University of Science and Technology, Irbid, JOR; 4 Department of Pediatrics, King Abdullah University Hospital, Ar Ramtha, JOR

**Keywords:** early onset inflammatory bowel disease, cmv, ebv, familial mediterranean fever, chronic granulomatous disease

## Abstract

The association between primary immunodeficiencies and autoinflammatory disorders has been popularized over the past decade. In this report, we illustrated the co-infection of cytomegalovirus (CMV) and Epstein-Barr virus (EBV) in a three-year-old Jordanian male patient with an extremely rare variant of the *CYBB* gene (c.125C>G, p.Thr42Arg) associated with chronic granulomatous disease (CGD) coexisting with familial Mediterranean fever (FMF). CGD and FMF co-existence induced early-onset inflammatory bowel disease mainly resembling Crohn’s disease.

## Introduction

In disorders of the immune system, autoimmunity and immunodeficiency are on the opposite ends of the spectrum. Despite the long belief of them being mutually exclusive, a developing foundation of their intricate interaction is being established, especially in the era of genetic analysis [1].

Chronic granulomatous disease (CGD) is a primary immunodeficiency affecting one in 250,000 individuals and is attributed to phagocyte malfunction in the domains of autophagy, apoptosis, and neutrophil extracellular traps [2,3]. Increased vulnerability to severe bacterial and fungal infections is a hallmark of the CGD course [4]. Unfortunately, neutrophils of CGD patients are deficient in the enzyme nicotinamide adenine dinucleotide phosphate (NADPH) oxidase, which consists of cell membrane-bound and cytoplasmic domains as in gp91phox, p22phox, p40phox, p47phox, and p67phox. It is primarily responsible for producing superoxide, which is utilized to kill phagocytosed organisms [3,5]. There are several mutations reported in the literature for CGD; the commonly known ones are CYBB/gp91phox, NCF1/p47phox, and CYBA/p22phox. Almost 70% of patients with CGD have a mutated *CYBB* gene, which is found on the X-chromosome, coding for cytochrome b-245 beta subunit [3,5,6]. Additionally, it has been shown that some described mutations result in an atypical form of the disease such as NCF4/p40phox deficiency, which is a mild form of CGD, though lacking invasive bacterial or fungal infections [2,5].

We present a case report of a patient with an atypical form of CGD co-existing with familial Mediterranean fever (FMF) complicated with Epstein-Barr virus (EBV) and cytomegalovirus (CMV) viremia.

## Case presentation

A four-month-old male infant, who was delivered by cesarean section at term with an uncomplicated neonatal period, presented to the emergency department with a documented fever (39-39.5℃) for three weeks associated with remarkable hypoactivity and sweating. The patient was first evaluated in a peripheral hospital regarding the same complaint and was treated conservatively as a case of upper respiratory tract infection. The same incidence recurred three times which implied further investigation revealing typical findings of meningitis in cerebrospinal fluid (CSF) analysis. The patient was admitted and treated with ceftriaxone and vancomycin for 10 days. Despite the non-resolving fever, he was discharged and readmitted with the same presentation.

Septic workup was negative. An abdominal computed tomography (CT) scan revealed hepatomegaly and a suspicious abdominal mass that resolved with antimicrobial therapy (piperacillin/tazobactam, vancomycin, and fluconazole). Complete blood count showed microcytic hypochromic anemia (hemoglobin: 8.5 g/dL (reference range: 10.5-14 g/dL), hematocrit: 26.9% (reference range: 32-42%), mean cell volume: 74.6 µm^3^ (reference range: 72-88 µm^3^)) with mild thrombocytosis (431.0×10^3^/mm^3^ (reference range: 150-450×10^3^/mm^3^)) and leukocytosis (17.7×10^3^/mm^3^ (reference range: 6-17×10^3^/mm^3^)). The cellular differential showed no neutropenia or lymphopenia. The chemistry panel and liver enzymes were normal. An elevated level of lactate dehydrogenase (LDH), 991.0 U/L (reference range: 170-580 U/L), was observed in association with high levels of ferritin (292.8 ng/mL (reference range: 50-200 ng/mL)), and erythrocyte sedimentation rate (ESR) (65 mm/h (reference range: 3-13 mm/h)). Direct Coombs test was positive with normal reticulocyte count (2.4%). The extractable nuclear antigen (ENA) panel was negative for anti-Sjögren's-syndrome-related antigen A (anti-SSA), anti-Sjögren's-syndrome-related antigen B (anti-SSB), anti-scleroderma (Scl)-70, anti-Smith, anti-Jo-1, and anti-ribonucleoprotein (anti-RNP). In addition, rheumatoid factor, anti-nuclear antibody, and anti-cyclic citrullinated peptide (CCP) were all negative. Serology testing for toxoplasmosis, rubella, parvovirus B19, human simplex virus 2 (HSV-2), CMV, and EBV didn’t yield any significant findings associated with negative hepatitis panel. Urine and stool analyses and cultures were non-remarkable, without evidence of rotavirus gastroenteritis.

Since then, the patient experienced intermittent high-grade fever attacks that lasted five to six days, almost every two weeks, which were responsive to antipyretic agents. Two years after the initial presentation, when the patient was two and a half years old, he presented with a fever (39.0℃) of five days associated with vomiting, hypoactivity, and decreased oral intake. The same pattern was seen in the vital signs and basic laboratory investigations as the initial visit, except for a negative direct Coombs test. Blood cultures were negative. This time, on the contrary, serology testing for CMV was positive, which was later confirmed with CMV polymerase chain reaction (PCR) testing. EBV PCR testing was positive as well. He was treated with imipenem, vancomycin, ganciclovir, and IV immunoglobulin (IVIG). Consequently, the patient became afebrile. Pathological description of bone marrow smear appeared unremarkable. The diagnosis of tuberculosis was eliminated with the negative Ziehl-Neelsen stain, and QuantiFERON-TB Gold (QIAGEN, Hilden, Germany) besides no growth in the corresponding cultures. Likewise, *Brucella* cultures were negative. Flow cytometry analysis was utilized to evaluate the presence of cell adhesion integrins as suspicion of leukocyte adhesion deficiency (LAD) was raised. Almost 99% of neutrophils and monocytes were expressing CD11a, CD11b, and CD18 omitting LAD diagnosis. Abdominal ultrasonography (U/S) demonstrated only mild splenomegaly with no focal lesions, the long axis of the liver measured 10.53 cm. Accordingly, the patient was discharged.

**Figure 1:**
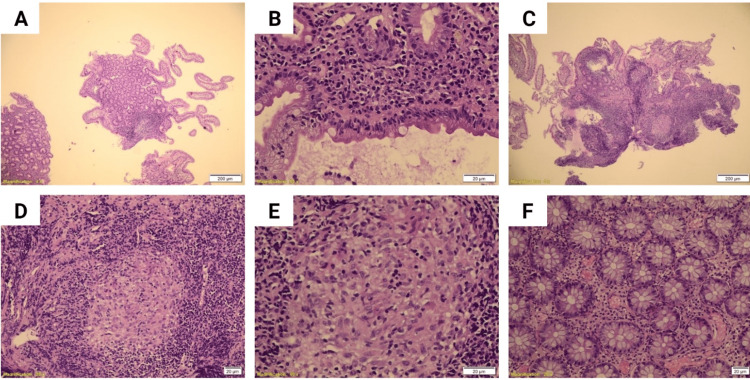
Duodenal mucosa with focal blunting of villous architecture with increase in intraepithelial lymphocytes (30L/100 enterocytes) (A, B) and the lamina propria shows increase in neutrophils and cryptitis (C, D, E). Colon mucosa with preserved crypt architecture and no significant pathologic changes (F).

When the patient was three yers old, he was admitted again with the same complaint along with severe atypical persistent diarrhea. An esophagogastroduodenoscopy (EGD) and colonoscopy were performed. Grossly EGD revealed elements of gastritis without modularity or ulceration, as well as duodenal hyperemia. Colonoscopy, on the other hand, showed perianal skin tags with old, healed fissures; the colon showed areas of loss of vasculature markings with areas of hyperemia and ulcers alternating with areas of normal mucosa. The rectosigmoid, cecum, and terminal ileum were normal with no definitive pathology identified. Multiple biopsies from the stomach, duodenum, colon, and terminal ileum revealed blunting of the villous architecture with increased intraepithelial lymphocytes (30 lymphocytes per 100 enterocytes) in the duodenum (Figure 1A-1B). The lamina propria showed an increase in acute and chronic inflammatory cell contents as well (including lymphocytes, neutrophils, plasma cells, and eosinophils) (Figure 1C-1E). Remarkably, the terminal ileum had a submucosal granuloma with a giant cell reaction with no evidence of infectious microorganisms, dysplasia, or malignancy (Figure 1C-1E), while colonic examination revealed a mild non-specific increase in chronic inflammatory cell content of the lamina propria (including lymphocytes, eosinophils, and plasma cells) (Figure 1F).

The recurrent unexplained fever spikes drew attention to the diagnosis of FMF, which was confirmed by *MEFV* gene PCR testing. Unfortunately, FMF did not explain the clinical course of recurrent infections, which rendered the need for further investigation. Whole exome sequencing (WES) confirmed the diagnosis of FMF with a heterozygous autosomal recessive mutation in the *MEFV* (NM_000243.2:c.2082G>A, NP_000234.1:p.Met694Ile) with a ClinVar ID of VCV000002539. An astonishing and surprising rare co-mutation in the *CYBB* gene inherited in X-linked fashion suggested the diagnosis of CGD (NM_000397.4:c.125C>G, NP_000388.2:p.Thr42Arg).

**Figure 2:**
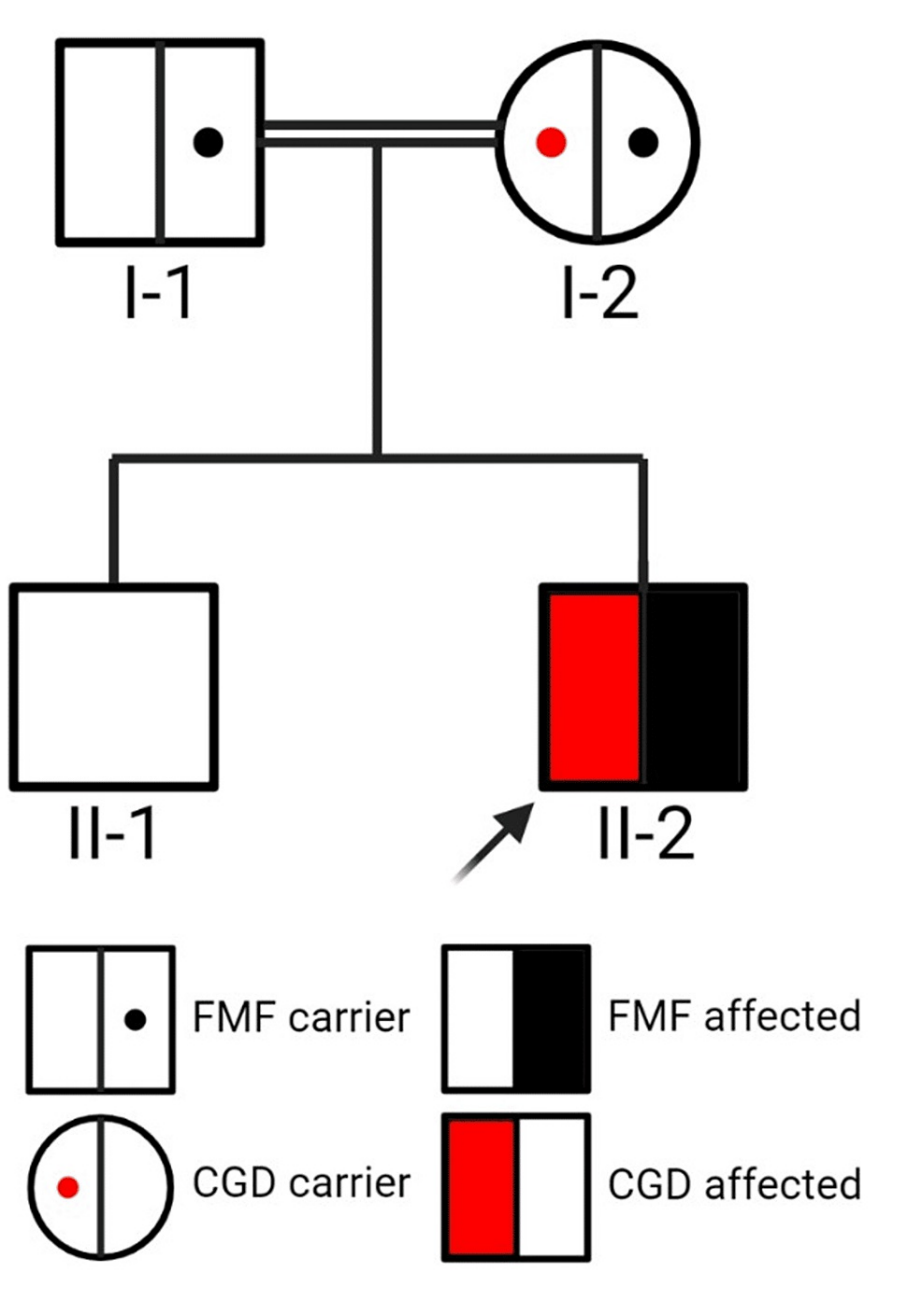
Family pedigree. CGD: chronic granulomatous disease; FMF: familial Mediterranean fever

**Figure 3:**
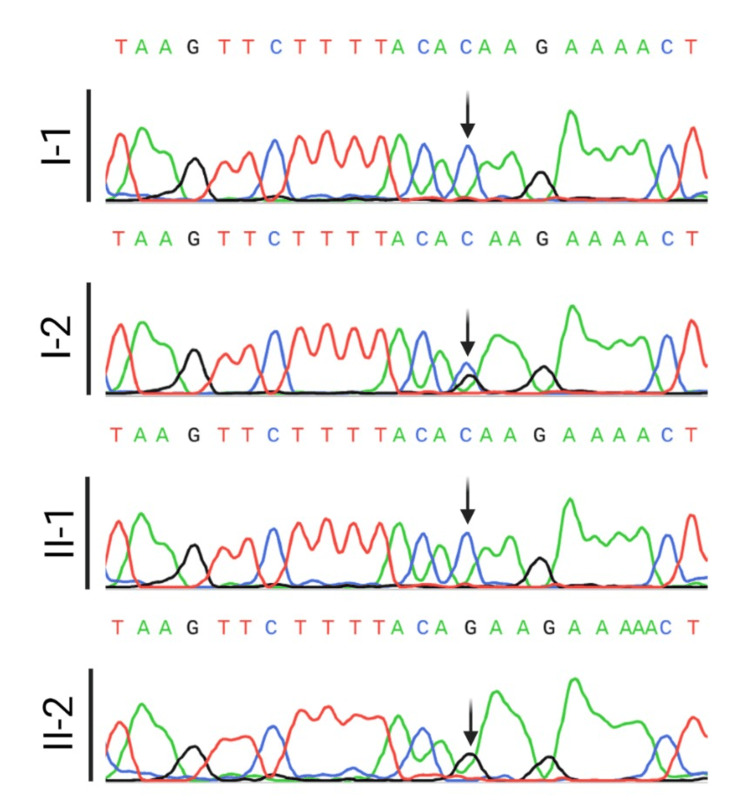
Sanger sequencing chromatogram of the CYBB gene.

This mutation was not reported in the gnomAD v2.1.1 dataset and silico prediction tools and conservation analysis predicted that this variant was probably damaging to the protein structure/function (REVEL: 0.801). Both parents were consanguineous (cousins) as shown in Figure 2. The GCD mutation reported is a missense mutation in the *CYBB* gene leading to Thr42Arg substitution with an unknown subtype (X91?); it has been previously reported and tagged as “unpublished X-linked disease-causing mutation” [3]. Sanger sequencing was performed to assess the *CYBB* variant (Figure 3), confirming the WES results. The mother showed (G/C) heterozygosity with a normal sequence in the proband’s brother and father.

## Discussion

CGD patients and female carriers have a greater risk of developing autoimmune disorders [2]. This is consistent with what has been observed in our patient who had a concomitant FMF diagnosis. FMF is an autoimmune disease affecting primarily people of Mediterranean descent and is the most common hereditary autoimmune disease in the world [7]. This results from a mutation in the *MEFV* gene coding for pyrin, which is responsible for innate immunity and inflammasome component leading to exaggerated inflammatory response through uncontrolled production of interleukin-1 [7,8]. It is characterized by episodic fever attacks, arthritis, serositis, dermal manifestations, and undesirable consequential renal mutilations [8]. The non-specificity of symptoms, which can mimic numerous other disorders, particularly Crohn’s disease as both affect the digestive tract, makes the diagnostic process a challenging one [9]. This made this case unique since the non-specific symptoms of FMF along with the granuloma formation due to CGD created a vague picture of Crohn’s disease, resembling it on various levels embracing clinical, anatomical, and histological aspects. As illustrated in the colonoscopy, a submucosal granuloma with a giant cell reaction was found in the terminal ileum, which could be a manifestation of CGD due to excessive NF-ĸB and inflammasome activation resulting in the production of pro-inflammatory cytokines [2].

Early-onset pediatric inflammatory bowel disease is still a rare disease condition, despite a rising incidence seen globally [10,11]. Compared to ulcerative colitis, Crohn’s disease is more frequently identified in children over the age of six years [12]. Pediatric inflammatory bowel disease has well-established symptoms, the most prevalent of which are diarrhea, blood in the stool, and abdominal pain [13]. Additionally, atypical manifestations are frequent, especially in younger children. For instance, patients with early-onset presentations are more likely to exhibit solitary rectal bleeding. Almost 20% of children with Crohn's disease are known to experience growth failure, and up to half of children under the age of six, who are diagnosed with pediatric inflammatory bowel disease, are reported to experience failure to thrive [13-16]. Pancolitis is a distinctive attribute of early-onset Crohn's disease and ulcerative colitis [17]. The presentation of our patient did not fit the profile of early-onset inflammatory bowel disease, and hence, the need for whole exome sequencing was raised, and other differential diagnoses floated to the top instead.

## Conclusions

In this report; we presented an extremely rare co-existence of CGD and FMF in a three-year-old Jordanian male patient. The patient experienced another rare incidence of CMV and EBV co-infection associated with early-onset inflammatory bowel disease. We presented the diagnostic approach used in this clinical scenario alongside the therapeutic management plan.
